# Supplementary material for: MetaRibo-Seq measures translation in microbiomes
Source: Nat Commun. 2020 Jun 29;11:3268. doi: 10.1038/s41467-020-17081-z (PMC7324362; doi:10.1038/s41467-020-17081-z)
Supplement: Supplementary file 10 — Supplementary Data 7 [file 41467_2020_17081_MOESM10_ESM.zip › File2/Confidence_VeryHigh_Taxonomy/767_out.krona.html]

Javascript must be enabled to view this page.

members
magnitude
magnitudeUnassigned
count
unassigned
taxon
rank

767\_out

60

2
60
superkingdom

phylum
60
976

200643
60
class

171549
1
order

SRS078242\_contig\_number\_contig-100\_12921.153573
60

2005525
59
family

genus
16
375288
59

SRS011134\_contig\_number\_contig-100\_10680.243278SRS017521\_contig\_number\_21384SRS018656\_contig\_number\_11082SRS019787\_contig\_number\_5349SRS019910\_contig\_number\_7979SRS022609\_contig\_number\_contig-100\_9684.210736SRS024435\_contig\_number\_contig-100\_8079.201295SRS024625\_contig\_number\_21164SRS048060\_contig\_number\_3669SRS048870\_contig\_number\_contig-100\_8431.104513SRS050925\_contig\_number\_16221SRS052027\_contig\_number\_contig-100\_17522.84312SRS056273\_contig\_number\_13549SRS064557\_contig\_number\_11929SRS1041145\_contig\_number\_13471SRS893341\_contig\_number\_16037

469591
1

SRS049900\_contig\_number\_14909
species

1947048
1

SRS016095\_contig\_number\_11334
species

823

SRS011239\_contig\_number\_15962SRS014313\_contig\_number\_4300SRS015369\_contig\_number\_contig-100\_3562.47142SRS016095\_contig\_number\_1943SRS016267\_contig\_number\_7695SRS016989\_contig\_number\_contig-100\_6364.84482SRS018351\_contig\_number\_18438SRS019496\_contig\_number\_12274SRS043768\_contig\_number\_16762SRS045195\_contig\_number\_1393SRS049712\_contig\_number\_contig-100\_612.108494SRS050974\_contig\_number\_contig-100\_930.931SRS053087\_contig\_number\_contig-100\_3289.3290SRS058145\_contig\_number\_1928SRS058723\_contig\_number\_7330SRS064232\_contig\_number\_contig-100\_1317.1318SRS064645\_contig\_number\_231SRS065397\_contig\_number\_3362SRS075078\_contig\_number\_10254SRS076929\_contig\_number\_20955SRS077194\_contig\_number\_21259SRS077194\_contig\_number\_22252SRS077335\_contig\_number\_5157SRS098655\_contig\_number\_11075SRS100021\_contig\_number\_4360SRS1041129\_contig\_number\_1825SRS1041130\_contig\_number\_contig-100\_6539.36332SRS1041134\_contig\_number\_15028SRS104693\_contig\_number\_10431SRS105082\_contig\_number\_22848SRS1055049\_contig\_number\_13802SRS1055056\_contig\_number\_6201SRS140492\_contig\_number\_32869SRS143876\_contig\_number\_1023SRS146813\_contig\_number\_4376SRS147653\_contig\_number\_contig-100\_1788.52921SRS148874\_contig\_number\_3945SRS149075\_contig\_number\_17665SRS893380\_contig\_number\_45SRS893383\_contig\_number\_contig-100\_7445.157499SRS971275\_contig\_number\_44054
41
species
